# Etiology, Predisposing Factors, Clinical Profile, Diagnosis, Management, Outcome, and Way Forward in Pulmonary Embolism at a Tertiary Care Center in East India

**DOI:** 10.7759/cureus.72000

**Published:** 2024-10-21

**Authors:** Tapan Kumar, Mandar M Shah, Aparna Prajapati

**Affiliations:** 1 Cardiology, Tata Main Hospital, Jamshedpur, IND; 2 General Medicine, Tata Main Hospital, Jamshedpur, IND

**Keywords:** acute chest pain, anticoagulation, pulmonary embolism, pulmonary hypertension, thrombolytic therapy

## Abstract

Background: Pulmonary embolism (PE) is a quite common condition and is a potentially lethal form of venous thromboembolic phenomenon associated with multiple inherited and acquired risk factors. Despite improvements in diagnostic techniques, delays in recognition and treatment are still common, contributing to increased morbidity and mortality. This study aimed to assess the time from the onset of symptoms to hospital arrival and to analyze delays in diagnosing and initiating treatment for patients with high and intermediate-to-high risk PE.

Materials and methods: A retrospective analysis was performed on 23 patients with high and intermediate-to-high risk PE admitted to a tertiary hospital from January 2021 to December 2023. Patients with pre-existing heart or lung conditions, low-risk PE, or those without follow-up were excluded. Routine blood tests, D-dimer measurement, 2D echocardiography, and CT pulmonary angiography (CTPA) were used to diagnose PE. Thrombolysis with streptokinase was administered in confirmed cases, and patient outcomes were monitored post-hospitalization.

Results: The study included 23 patients, with a mean age of 47.1 years, 65% of whom were male. The most frequent symptom was shortness of breath (78%), followed by chest pain (13%) and syncope (9%). Elevated D-dimer levels were found in 96% of patients, and right ventricular overload was seen in all cases through echocardiography. CTPA confirmed PE in 91% of patients. Significant delays were observed in both hospital arrival and in-hospital diagnosis, with an average delay of more than seven hours before treatment began. Early diagnosis followed by thrombolysis led to favorable outcomes, while delayed diagnosis resulted in poorer outcomes, including residual symptoms and right ventricular dysfunction.

Conclusion: Early diagnosis and treatment of PE greatly enhance patient outcomes, especially with timely thrombolysis. Echocardiography should be utilized promptly in emergency settings to facilitate early treatment. Quick recognition of PE, particularly in patients with risk factors, is essential to reduce mortality and long-term complications.

## Introduction

Pulmonary embolism (PE) is a serious condition with potentially fatal consequences, ranking as the third most critical cardiovascular syndrome worldwide, following myocardial infarction and stroke [1]. Precise epidemiological figures are elusive, but it is estimated that PE affects around 60-70 individuals per 100,000 [2]. Due to nonspecific symptoms, vague signs, and limited bedside diagnostic capabilities, numerous cases of PE likely go undetected posing a challenge in accurately determining its true incidence.

Clinicians frequently encounter difficulty in diagnosing PE due to the predominantly nonspecific nature of its signs and symptoms. Despite advancements in diagnostic technology, there is not a single point-of-care test that definitively confirms the diagnosis [3]. Sudden onset of unexplained dyspnoea or rapid breathing should raise prompt suspicion of PE. Utilizing appropriate available diagnostic modalities, including electrocardiogram, chest X-ray, 2D echocardiography, and CT pulmonary angiography (CTPA) as soon as suspicion arises is crucial for timely diagnosis and management.

According to the European Society of Cardiology (ESC) 2019 guidelines, PE can be categorized [4] into low-risk, intermediate-risk (both high and low), and high-risk groups based on clinical presentation and hemodynamic severity. Data indicate that high-risk PE represents 5-10% of all cases and contributes significantly to poor outcomes in terms of both morbidity and mortality, particularly if treatment is delayed or inadequate. Even with treatment, mortality rates for diagnosed high- and intermediate-risk PE range from 3% to 8%, escalating to 30% if treatment is delayed [5]. Hence, delayed diagnosis and late thrombolysis exacerbate morbidity and mortality underscoring the importance of prompt diagnosis and aggressive treatment [6]. Early intervention not only aids in swiftly normalizing hemodynamic parameters and right ventricle function but also reduces morbidity and mortality [7]. Low-risk PE diagnosis is frequently missed due to mild symptoms, but more critically, even high- or intermediate-risk cases are often not detected on time.

Given this context, we initiated this study with two primary objectives: (1) to evaluate the duration of onset of symptoms to the patient's arrival at the emergency department and (2) to analyze the delay in diagnosis and commencement of appropriate treatment following hospitalization. Additionally, we present our findings regarding the clinical characteristics, diagnosis, management, outcomes, and future directions for patients with symptomatic PE in tertiary care facilities.

## Materials and methods

We conducted a retrospective analytical examination of patients diagnosed with acute intermediate-to-high-risk and high-risk pulmonary thromboembolisms who were admitted to our hospital between January 2021 and December 2023. All patients underwent routine blood investigations, chest X-rays, D-dimer tests, and 2D echocardiography. CTPA was done for 21 patients; two patients could not be subjected to CTPA due to acute hemodynamic instability.

Patients with pre-existing cardiac conditions or lung disorders were not included in the study. Additionally, individuals with low-risk pulmonary embolism, which typically follows a benign course were also excluded as our primary interest lay in examining the acute and long-term outcomes of high-risk cases. Furthermore, patients who did not undergo follow-up were excluded from the analysis.

Patients diagnosed with pulmonary embolism were categorized [3] into three groups: high risk if they displayed signs of hemodynamic compromise (characterized by a systolic blood pressure below 90 mm Hg), intermediate risk if echocardiography revealed right ventricular overload or failure without systemic compromise, and low risk if they did not exhibit any of these features.

Echocardiographic parameters for evidence of right ventricular overload/failure increased in RV/LV (right ventricle/left ventricle) dimension ratio, hypokinesia or akinesia of the RV free wall, "D-shape" LV cavity, pulmonary artery dilation, tricuspid regurgitation (TR) of > 2.8 m/sec, reduced inferior vena cava fluctuations with respiration (<40%) [8].

Thrombolytic agents approved by the FDA [9,10] for treating PE include streptokinase, urokinase, and alteplase. In our treatment protocol, we administered streptokinase to our patients at a dose of 250,000 units as a loading dose over 30 minutes, followed by 100,000 units per hour for the next 12-24 hours adjusted according to the individual's hemodynamic response.

We monitored all patients throughout their hospitalization, as well as on the 7th-day post-discharge and then again at one month and six months thereafter. This monitoring aimed to track clinical progress and assess echocardiographic parameters such as pulmonary arterial hypertension and right ventricular dysfunction.

Statistical analysis for this study was conducted using Statistical Product and Service Solutions (SPSS, version 27; IBM SPSS Statistics for Windows, Armonk, NY), primarily focusing on descriptive statistics. Continuous variables, such as age and symptom duration, were reported as mean values with standard deviations. Categorical variables, such as predisposing factors and clinical symptoms, were presented as frequencies and percentages. The association between clinical severity and delays in treatment was evaluated using the chi-square test for categorical data, with statistical significance set at a p-value of less than 0.05. Data were also stratified based on time delays to examine differences in outcomes. Due to the small sample size, no advanced multivariate analysis was performed.

## Results

Patient demographics and exclusions

**Figure 1:**
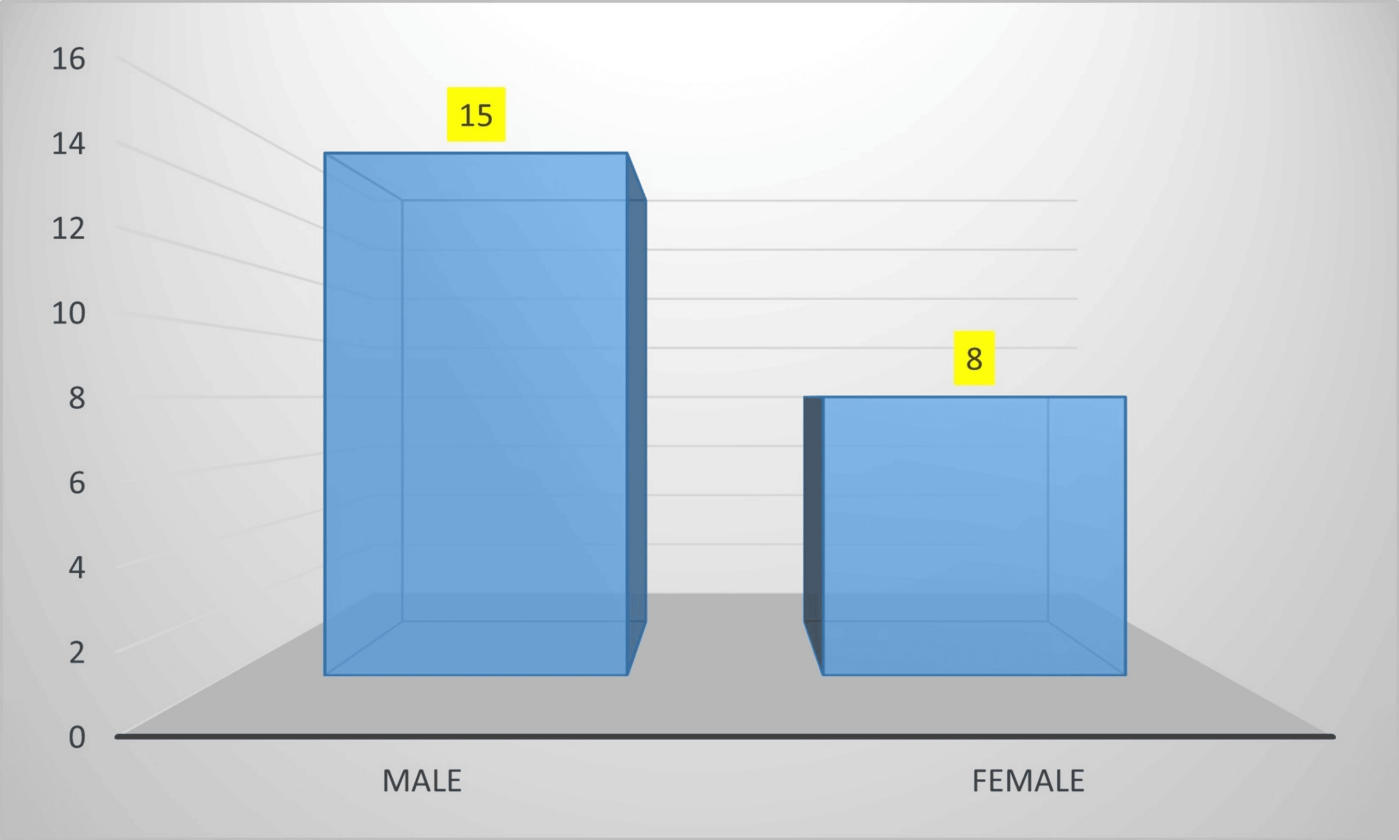
Gender distribution in the study subjects X axis: Gender; Y axis: Number of patients

We analyzed a total of 39 patients diagnosed with acute PE. Sixteen patients were excluded either due to not fulfilling our inclusion criteria or lacking proper follow-up. Following these exclusions, a total of 23 patients who met our criteria were included in the study. The mean age was 47.1 years. Among 23 patients, 15 (65%) were males, and eight (35%) were females, as shown in Figure 1.

Laboratory findings

The D-dimer level was elevated in 22 (96%) out of 23 patients. The findings of routine blood tests were non-specific. Chest X-ray was not confirmatory of PE in any of our patients.

Predisposing factors for PE

There were various predisposing factors (Table 1) for the development of acute PE in the study. Most common was prolonged immobilization (12, 52%) patients, of which three (13%) had a history of long air travel, three (13%) had long road travel, two (9%) were shopkeepers, and four (17%) patients had a history of continuous long hours of computer work for approximately 10 hours, followed by malignancy (5, 22%), of which two patients had carcinoma breast, two had carcinoma stomach, one had colon carcinoma and one nephrotic syndrome (4%), two patients (9%) had anticoagulation factor C deficiency; and post-operative, three (13%) had recent orthopedic surgery and not on any anticoagulation.

**Table 1:** Predisposing factors

Predisposing factors	Numbers of patients (%)
History of immobilization	12 (52)
Malignancy	5 (22)
Protein C or S deficiency	2 (9)
Nephrotic syndrome	1 (4)
Post-operative	3 (13)

Presenting symptoms

**Figure 2:**
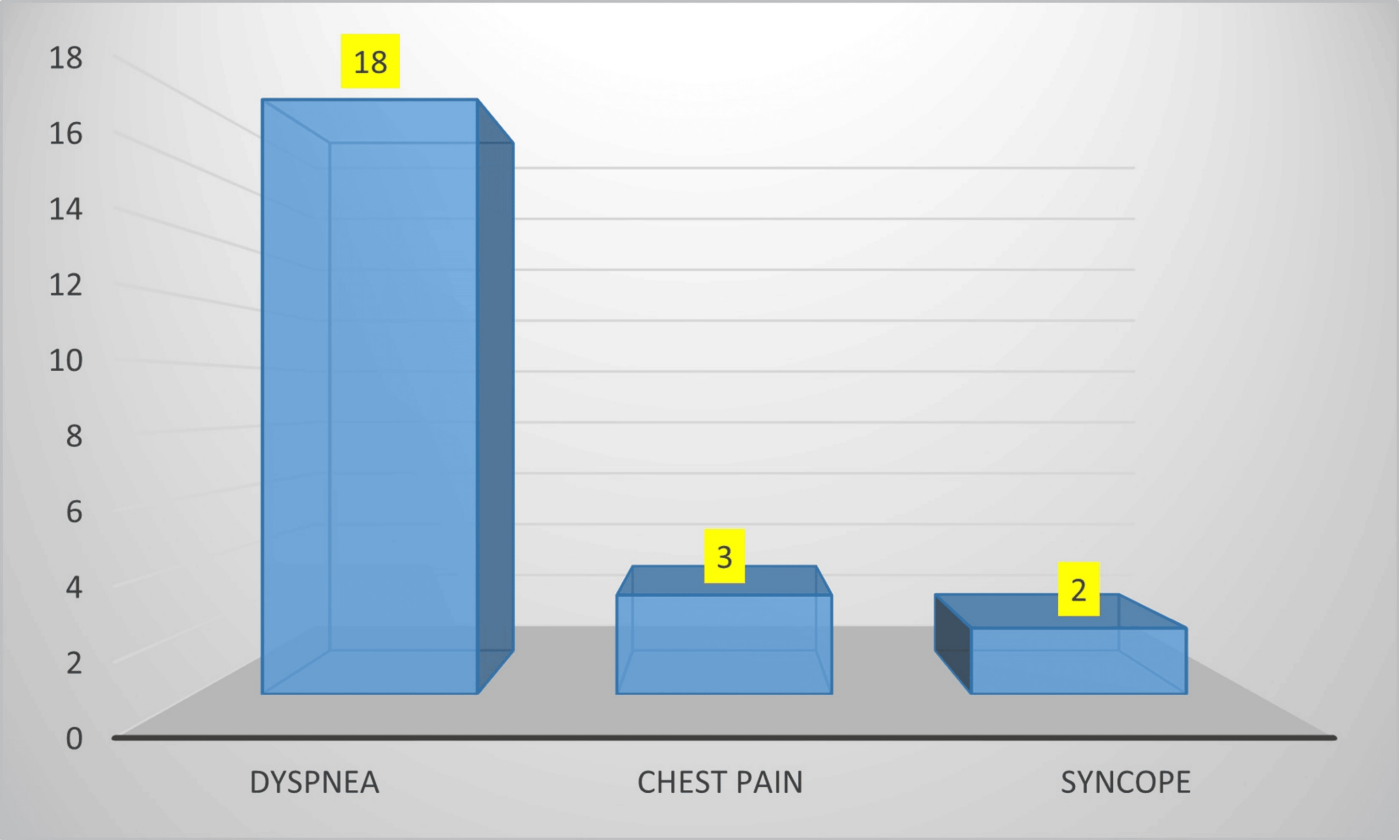
Presenting symptoms in the study subjects X axis: Presenting symptoms; Y axis: Number of patients

The commonest presenting symptom was dyspnoea in 18 patients (78%), followed by chest pain in three patients (13%) and syncope in two patients (9%) (Figure 2). The mean duration of onset of symptoms to hospitalization was 16 hours.

Clinical severity

Clinical severity has been represented in Table 2. Two patients presented with life-threatening symptoms such as syncope, severe shock, or frank cardiac arrest; seven patients had hypotension but without cardiogenic shock; and the other 14 had severe right ventricular dysfunction.

**Table 2:** Incidence of individual clinical manifestations based on severity

Clinical presentation	Numbers of patients
Syncope, severe shock or cardiac arrest	2 (9%)
Hypotension without signs of shock	7 (30%)
Hemodynamic stability, but echocardiographic signs of right ventricular dysfunction	14 (61%)

Electrocardiographic findings

**Figure 3:**
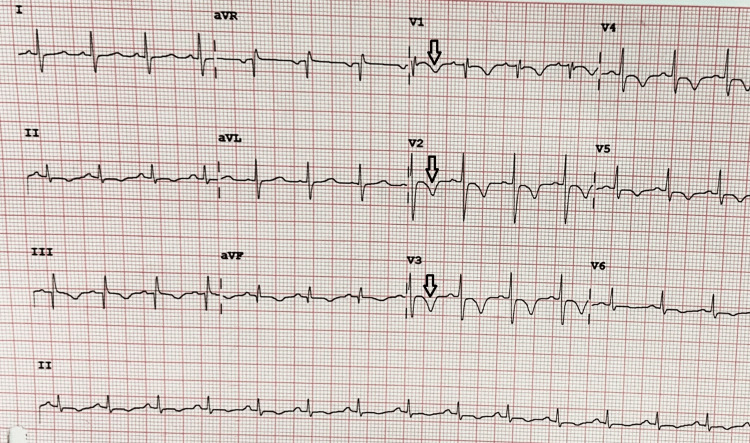
ECG representing all typical features of PE, including sinus tachycardia, RV strain, and S1Q3T3 Arrows showing the T inversion

Sinus tachycardia (91.4%), followed by RV strain pattern (65.7%) and then S1Q3T3 pattern (34.2%) pathognomonic of right heart strain and right bundle branch block (RBBB) (20%), were electrocardiographic findings. This ECG in Figure 3 represents all typical features of PE, including sinus tachycardia, RV strain, and S1Q3T3. 

Echocardiographic findings

2D Echocardiographic findings are mentioned in Table 3, which showed features of right ventricle overload in almost all patients as well as other features of RV strain in almost 90% of patients.

**Table 3:** Echocardiographic findings

Echocardiographic parameter	Numbers of patients (%)
Increase in RV/LV dimension ratio	23 (100)
Hypokinesia or akinesia of the RV-free wall	19 (78)
"D-shape" of the left ventricle	19 (78)
Pulmonary artery dilation	15 (62)
Tricuspid regurgitation of > 2.8 m/sec	15 (62)

**Figure 4:**
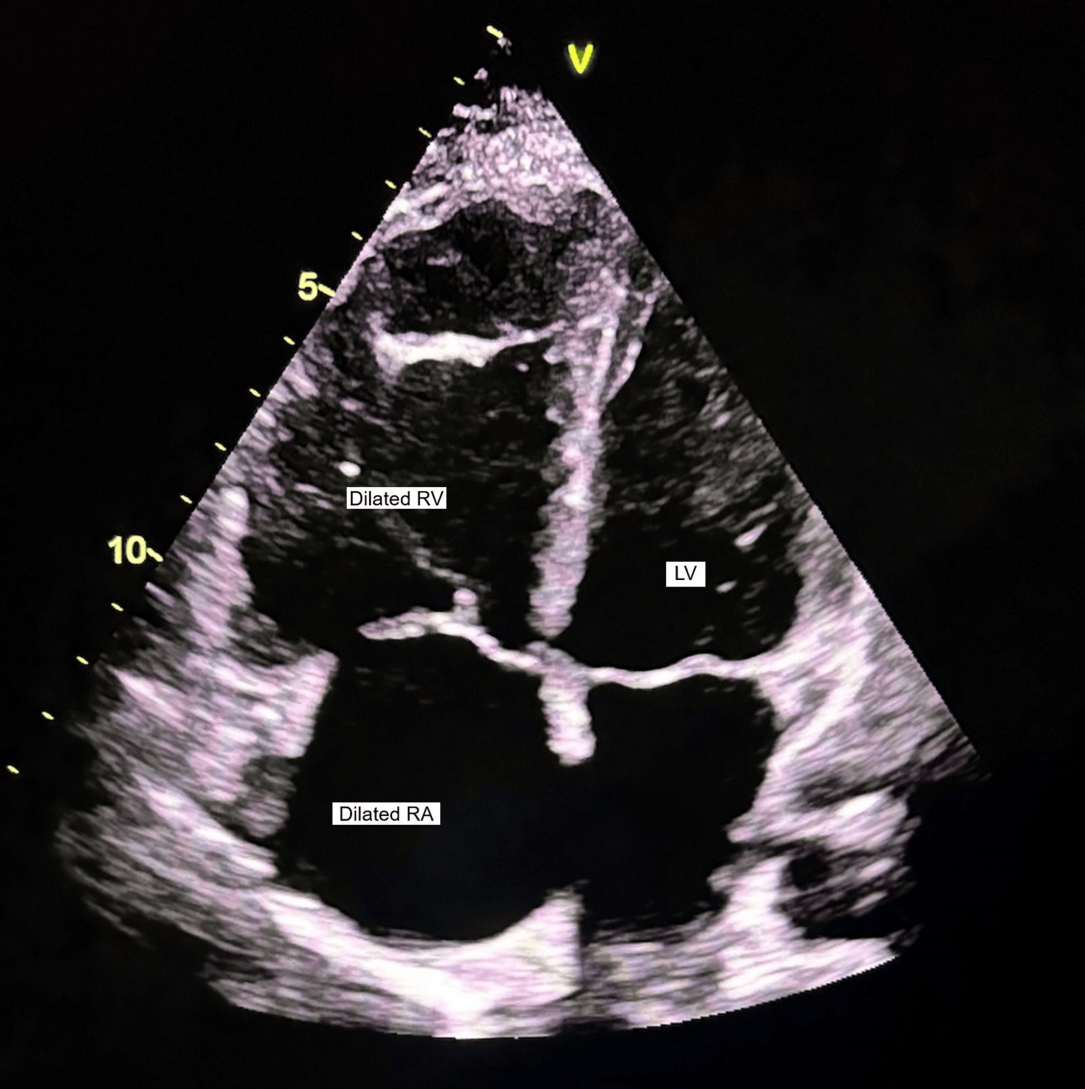
Increased RV/LV dimension in systole RV: Right Ventricle; RA: Right Atrium; LV: Left Ventricle

**Figure 5:**
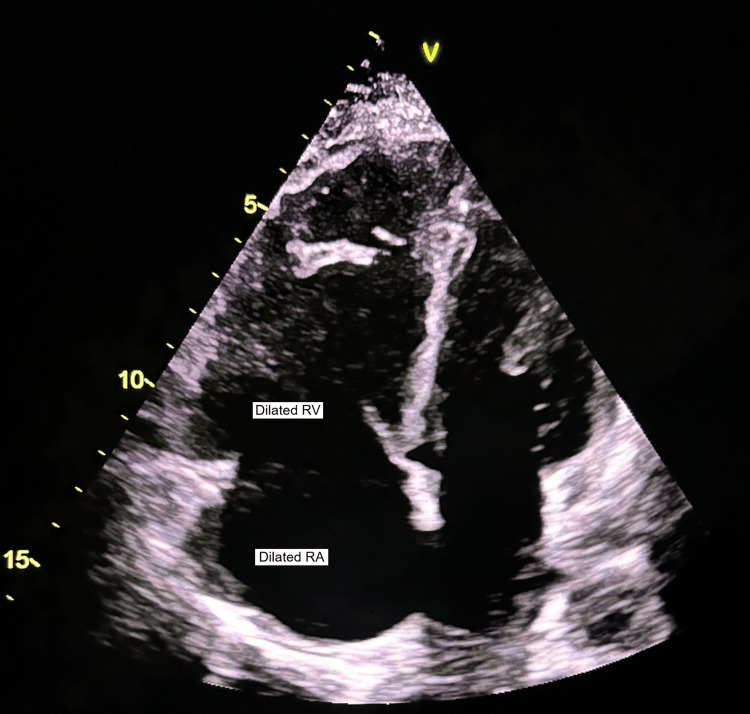
Increased RV/LV dimension in diastole RV: Right Ventricle; RA: Right Atrium; LV: Left Ventricle

Figures 4-5 show the markedly dilated RA and RV with increased RV/LV dimension and D-shaped left ventricle.

**Figure 6:**
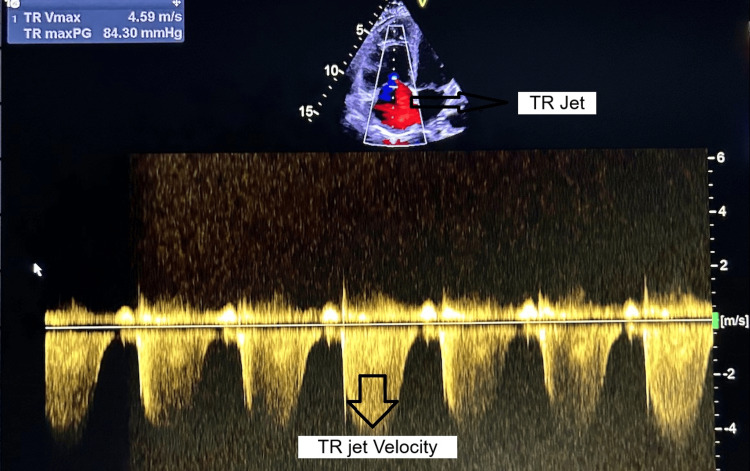
Severe tricuspid regurgitation and pulmonary artery systolic pressure (PASP) TR: Tricuspid regurgitation Asterisk showing TR jet and TR jet velocity

Figure 6 shows significant TR with raised pulmonary artery systolic pressure (PASP).

**Figure 7:**
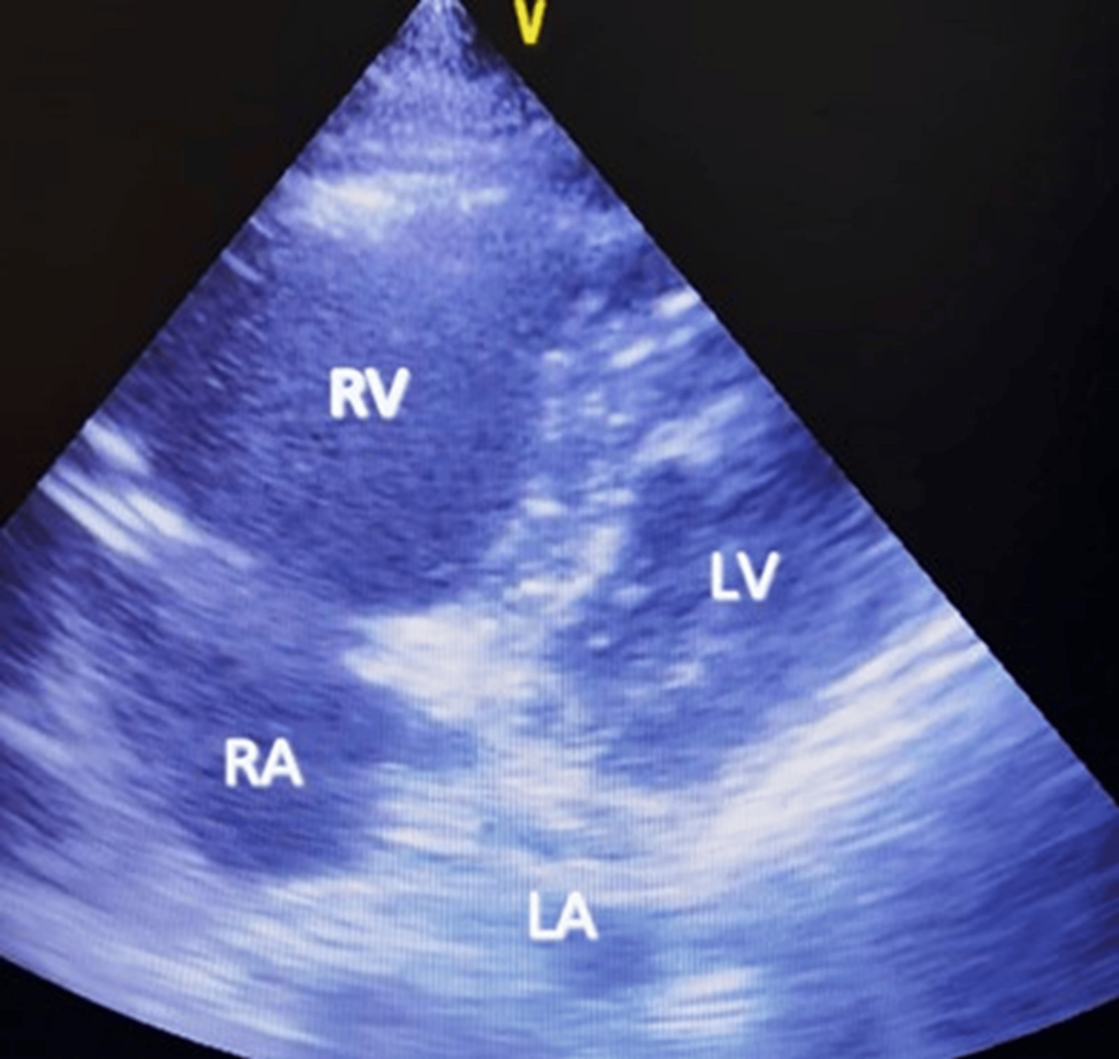
Bedside echo image of massive PE PE: Pulmonary Embolism; RV: Right Ventricle; RA: Right Atrium; LV: Left Ventricle; LA: Left Atrium

Figure 7 displays a bedside echocardiogram of a patient who presented with a history of syncope at home and was brought to the emergency room in a gasping condition. A rapid bedside echocardiogram revealed a significantly enlarged right atrium and ventricle encroaching upon the entire left atrium and ventricle, which was adequate to confirm the diagnosis of pulmonary embolism in conjugation with sudden, unexplained dyspnea and hemodynamic instability.

Pulmonary angiography findings

**Figure 8:**
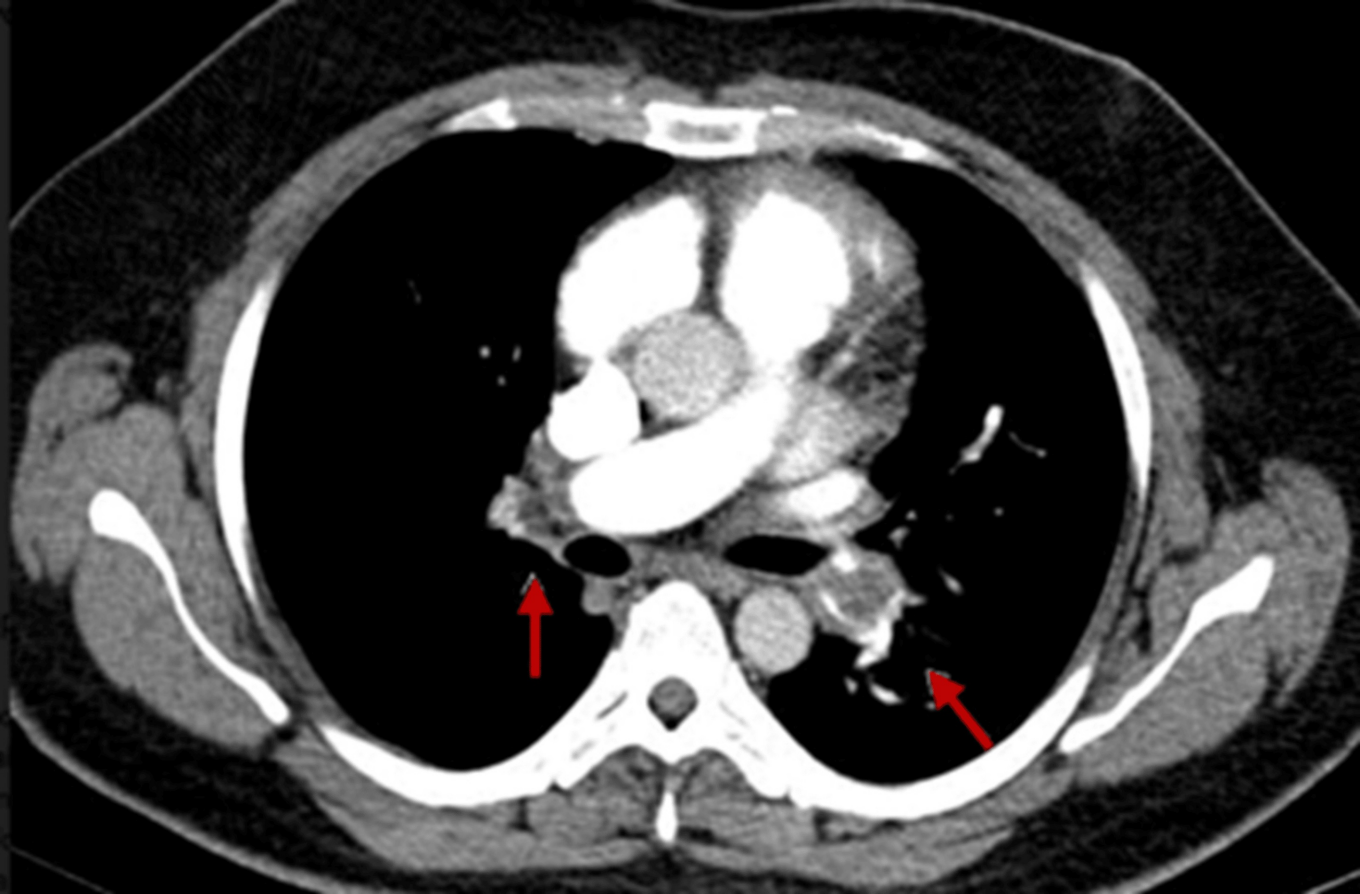
Involvement of both PA PA: Pulmonary artery Arrows showing pulmonary artery

**Figure 9:**
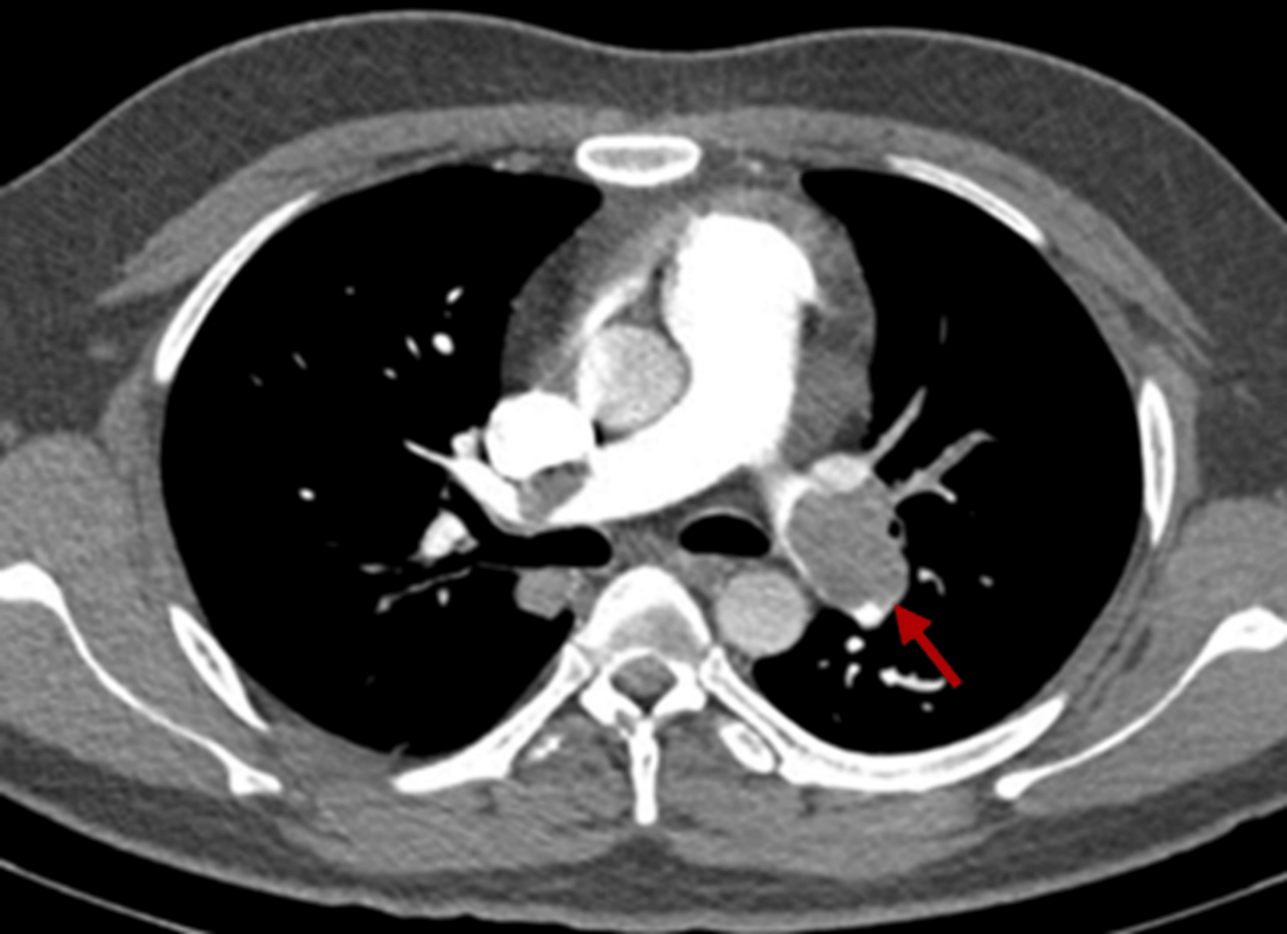
Primary involvement of RPA RPA: Right pulmonary artery The arrow showing the right pulmonary artery

CT pulmonary angiography (Figures 8-9) was done in all, except in two patients, to confirm the diagnosis. One of these two patients had in-hospital syncope and was treated successfully, and others also had syncope at home with the symptoms, signs, and echocardiographic features typical of PE treated successfully without residual sequelae. In other 21 (87%) patients, three (13%) had near complete occlusion of both left and right pulmonary artery, eight (34%) had occluded right pulmonary artery, three (13%) had occluded left pulmonary artery, and the remaining seven (30%) had involvement of multiple sub-segmental arteries.

Delay in diagnosis and treatment

There was a significant delay in all the patients before specific management was started. The delay was not only from the patient part, as mentioned in Table 4, but also there was a significant delay in in-hospital diagnosis after hospitalization noted in Table 5. Therefore, delay in initiation of treatment has two components first is a delay in arrival to the hospital, and second is an in-hospital delay in diagnosis.

**Table 4:** Duration between the onset of symptoms and arrival at emergency

Numbers of patients (%)	Average delay in reaching the hospital (in hours)
4 (18)	3.75
11 (47)	7.36
3 (13)	11.2
5 (22)	17.4

**Table 5:** In-hospital delay in diagnosis

Numbers of patients (%)	Average in-hospital delay in diagnosis (in hours)
6 (26)	2.1
7 (31.5)	3.4
7 (31.5)	8.9
3 (13	7.1

The minimum delay was more than five hours, whereas, in some patients, it was even more than 24 hours. Based on time delay, both groups were further subdivided into four categories. Maximum patients were in group 2. The common reasons for in-hospital delay in diagnosis were the low index of suspicion, lack of expertise, and unavailability of echocardiography in an emergency and delay in arranging the confirmatory imaging modality CT pulmonary angiography.

## Discussion

PE poses a significant risk to life and presents a diagnostic puzzle for emergency clinicians. However, timely diagnosis and treatment can yield rewarding outcomes.

We examined 23 patients, with an average age at presentation of 47.1 years and a preponderance of males. However, in some other studies [10], the gender distribution was nearly equal.

PE occurs when a blood clot obstructs the pulmonary artery or its branches. However, in some cases, blockages can also be caused by substances other than blood clots, such as fat from a fractured long bone, fragments of a tumor, or air bubbles. However, in our series, all the patients had thromboembolism [10].

The clinical presentations varied but dyspnea was the most common symptom, accompanied by sinus tachycardia and tachypnea as the most frequent signs. Dyspnea was observed in 18 (78%) patients, while three (13%) presented with symptoms resembling acute coronary syndrome, and two (9%) experienced syncope.

The D-dimer assay [11] is a crucial blood screening test with a robust negative predictive value, effectively ruling out PE if negative. In our study, 22 patients had elevated D-dimer levels, providing strong supportive evidence. Additionally, electrocardiography (ECG) serves as another valuable bedside investigation. Among the patients, 21 (91%) displayed sinus tachycardia, 15 (64%) exhibited signs of right ventricular strain, and eight (26%) showed the S1Q3T3 pattern in conjunction with sinus tachycardia, all serving as indicators of PE.

Echocardiography serves as a rapid and crucial diagnostic tool for identifying right ventricular overload, indicated by features such as a dilated right atrium, enlarged right ventricle, and hypokinesia or dyskinesia of the right ventricular wall. Among all patients, there was an increase in the ratio of right ventricular to left ventricular dimensions, with 19 (83%) displaying hypokinesia of the right ventricular free wall and a D-shaped left ventricular cavity. Tricuspid regurgitation and dilation of the pulmonary artery were observed in 15 (65%) patients [12].

Thus, our study reemphasizes the fact that right ventricle involvement is a highly sensitive finding in echocardiography. Echocardiography also aids in excluding other significant differentials such as myocardial infarction and pericardial diseases. Here, again, we want to stress the fact that echocardiography should be done at the earliest, if possible, in an emergency for a better outcome.

CTPA stands as the gold standard for confirming the diagnosis. CTPA was performed in 21 (91%) patients, with the exception of two (9%) patients who were hemodynamically too unstable and thus did not allow sufficient time for the test. However, in these cases, the diagnosis of PE was confirmed through thorough history-taking, ECG, and echocardiographic findings, leading to judicious thrombolysis without delay. This diagnostic test not only confirms the diagnosis but also provides localization and assesses the extent of thrombus in pulmonary vessels aiding in patient prognosis [12].

Thrombolytic therapy was administered immediately after diagnostic confirmation, along with subsequent treatment involving low molecular weight heparin and non-vitamin K oral anticoagulants (NOACs). This serves as the primary management approach for high-risk PE cases.

Four patients who received timely thrombolysis within six hours of symptom onset showed excellent long-term outcomes with no residual right ventricular dysfunction. In 11 patients (48%), the diagnosis was delayed by more than 12 hours, resulting in suboptimal long-term results and residual symptoms such as dyspnea. The most concerning group consisted of eight patients (35%) whose diagnosis was delayed by more than 20 hours. Out of these eight patients, two patients (9%) succumbed to progressive right ventricular dysfunction over a two-year period, while the remaining six experienced significant symptoms related to right ventricular overload. Long-term anticoagulation with NOACs is preferred over conventional warfarin therapy due to its superior safety profile [12]. All our patients were given anticoagulation.

Limitations: This study is limited by its small sample size and single-center design, which may reduce the generalizability of findings. Additionally, the retrospective nature of the study may have introduced bias in data collection, and follow-up beyond six months was not conducted for all patients.

## Conclusions

PE is a life-threatening condition, but its outcomes can greatly improve with prompt diagnosis and aggressive treatment. Our first recommendation is for smaller medical centers with limited resources. In such settings, local physicians should promptly refer suspected patients to tertiary care centers for further evaluation and management. Secondly, in higher medical centers, it is imperative for the emergency team to have the necessary expertise to promptly diagnose this condition on-site. Thirdly, we want to emphasize the importance of clinicians considering PE as a potential differential diagnosis, especially when patients present with symptoms such as unexplained dyspnea or sinus tachycardia in the background of prolonged immobilization, recent surgery, or underlying carcinoma. In such cases, immediate echocardiographic examination should be performed in the emergency department, followed by CTPA if necessary, and thrombolytic therapy should be initiated promptly if indicated. Early diagnosis and treatment of this condition are crucial in preventing significant morbidity and mortality in patients.
